# Supplementary material for: Predation and fragmentation portrayed in the statistical structure of prey time series
Source: BMC Ecol. 2009 May 6;9:10. doi: 10.1186/1472-6785-9-10 (PMC2689204; doi:10.1186/1472-6785-9-10)
Supplement: Additional file 2 — Voles and related classes ODDox Documentation. ODDox documentation of the agent-based model (ALMaSS) applied by Hendrichsen et al. The documentation is started by activating main.html. [file 1472-6785-9-10-S2.zip › Vole_ODDox/functions_func_0x63.html]

ALMaSS ODDox: Class Members - Functions

- Main Page
- Related Pages
- Classes
- Files

- Alphabetical List
- Class List
- Class Hierarchy
- Class Members

- All
- Functions
- Variables

- a
- b
- c
- d
- e
- f
- g
- h
- i
- k
- l
- m
- n
- o
- p
- r
- s
- t
- u
- v
- w
- ~

### - c -

- CalculateCarryingCapacity()
  : Vole\_Base- CanFeed()
    : Vole\_Male- Catastrophe()
      : Population\_Manager
      , Vole\_Population\_Manager- CattleIsOut()
        : Farm- CattleIsOutLow()
          : Farm- CattleOut()
            : Farm- CattleOutLowGrazing()
              : Farm- CheckManagement()
                : TAnimal- CheckManagementXY()
                  : TAnimal- CheckRotationManagementLoop()
                    : Farm- CloseFile()
                      : probe\_data- CloseTheCIPEGridOutputProbe()
                        : Population\_Manager- CloseTheMonthlyRipleysOutputProbe()
                          : Population\_Manager- CloseTheReallyBigOutputProbe()
                            : Population\_Manager
                            , TPredator\_Population\_Manager- CloseTheRipleysOutputProbe()
                              : Population\_Manager
                              , TPredator\_Population\_Manager- ConventionalCattle()
                                : ConventionalCattle- ConventionalPig()
                                  : ConventionalPig- ConventionalPlant()
                                    : ConventionalPlant- ConvMarginalJord()
                                      : ConvMarginalJord- CopyMyself()
                                        : Vole\_Base
                                        , TAnimal- CreateObjects()
                                          : TPredator\_Population\_Manager
                                          , Vole\_Population\_Manager- CreateObjects\_Init()
                                            : Vole\_Population\_Manager- CropRotation()
                                              : CropRotation- CutToHay()
                                                : Farm- CutToSilage()
                                                  : Farm- CutWeeds()
                                                    : Farm

---

Generated on Thu Jan 22 14:13:45 2009 for ALMaSS ODDox by 
 1.5.6
